# Changes over lactation in breast milk serum proteins involved in the maturation of immune and digestive system of the infant

**DOI:** 10.1016/j.dib.2016.02.046

**Published:** 2016-02-27

**Authors:** Lina Zhang, Marita de Waard, Hester Verheijen, Sjef Boeren, Jos A. Hageman, Toon van Hooijdonk, Jacques Vervoort, Johannes B. van Goudoever, Kasper Hettinga

**Affiliations:** a Dairy Science and Technology, Food Quality and Design group, Wageningen University, The Netherlands; b Department of Paediatrics, VU University Medical Center, Amsterdam, The Netherlands; c Laboratory of Biochemistry, Wageningen University, The Netherlands; d Biometris-Applied Statistics, Wageningen University, The Netherlands; e Centre for BioSystems Genomics, Wageningen University, The Netherlands; f Academic Medical Center, Emma Children׳s Hospital, Amsterdam, The Netherlands

**Keywords:** Milk serum proteome, Gastrointestinal tract, Immune system, Protease inhibitors

## Abstract

Here we provide data from shot-gun proteomics, using filtered-aided sample preparation (FASP), dimethyl labeling and LC–MS/MS, to quantify the changes in the repertoire of human milk proteins over lactation. Milk serum proteins were analyzed at week 1, 2, 3 4, 8, 16, and 24 in milk from four individual mothers. A total of 247 proteins were identified, of which 200 proteins were quantified. The data supplied in this article supports the accompanying publication (Zhang et al., 2006) [1]. The mass spectrometry proteomics data have been deposited to the ProteomeXchange Consortium (Vizcaíno et al., 2016) [2] via the PRIDE partner repository with the dataset identifier PXD003465.

**Specifications Table**

**Table:** 

Subject area	Biology
More specific subject area	Lactation stage induced changes in the human milk proteome
Type of data	Raw LC/MSMS datafiles. Spreadsheets containing raw output from MaxQuant as well as filtered and annotated output.
How data was acquired	Filtered-aided sample preparation (FASP), dimethyl labeling followed by LC–MS/MS (Thermo Orbitrap-XL).
Data format	.RAW files (Thermo proprietary) and Excel files for data analysis output.
Experimental factors	Milk serum from week 1 until week 24 in lactation
Experimental features	Individual human milk serum samples were separated by ultracentrifugation, digested with trypsin (using FASP), labeled with dimethyl labeling, and analyzed by LC–MS/MS. The dimethyl ratios were determined for each peptide and normalized peptide ratios per protein were used for further data analysis.
Data source location	Samples were collected from women who gave birth at the obstetric department in VU University medical center (VUmc) in Amsterdam.
Data accessibility	The mass spectrometry proteomics data have been deposited to the ProteomeXchange Consortium [2] via the PRIDE partner repository with the dataset identifier PXD003465. An Excel document with data output is linked directly to this article.

**Value of the data**

• This data shows qualitative and quantitative variability of the human milk proteome, both between mothers and over lactation.

• A large number of proteins were identified, and a substantial proportion of those with altered abundance as lactation advances have functions associated with immunity and digestion.
• These data lead to a better understanding of the importance of human milk serum proteins in health and development of infants, which can be used as reference in improving infant formula.

## 1 **Data**

The mass spectrometry proteomics data have been deposited to the ProteomeXchange Consortium [2] via the PRIDE partner repository with the dataset identifier PXD003465. Data analysis output from MaxQuant is made available as Excel document, both as raw output as well as filtered and annotated output.

The raw MaxQuant output contains all the distinct proteins that were identified and quantified along with the number of peptides detected, and the dimethyl labeling ratios. The filtered and annotated output contains filtered proteins based on the identification criteria (At least two peptides per protein) and biological functions, determined by querying the GO database and Uniprot database. This filtered and annotated dataset is the basis of the accompanying publication “Changes over lactation in breast milk serum proteins involved in the maturation of immune and digestive system of the infant” [1].

## 2 **Experimental design, materials and methods**

### 2.1 Milk collection and sample preparation

Human milk samples were collected from women who gave birth at the obstetric department in VU medical center (VUmc) in Amsterdam. All women who delivered singleton term infants (gestational age 37–42 weeks) were eligible for this study. Since previous studies on distinct proteins showed a difference especially in early lactation [3], [4], samples were assessed weekly in the first month and every two months (at week 8, 16, and 24) thereafter. Approximately 5–10 mL was collected in a polypropylene bottle after one minute of pumping for every sample and stored at −18 °C immediately afterwards.

The separation of milk serum was performed according to a previous study [5]. The samples were centrifuged at 1500×g for 10 min at 10 °C (Beckman coulter Avanti J-26 XP centrifuge, rotor JA-25.15). The milk fat was removed and the obtained supernatant was transferred to the ultracentrifuge tubes followed by ultracentrifugation at 100,000*g* for 90 min at 4 °C (Beckman L-60, rotor 70 Ti). An aliquot of the skimmed milk was centrifuged at 100,000*g* for 60 min at 30 °C to pellet the casein micelles, and the clear supernatant (milk serum) was removed and stored at −20 °C.

### 2.2 Filtered-aided sample preparation (FASP)

Milk serum samples (20 μL), including samples of each time point and pooled samples per included women, were diluted in 100 mM Tris/HCl pH 8.0+4% SDS+0.1 M Dithiotreitol-DTT (SDT-lysis buffer ) to get a 1 μg/μL protein solution. Samples were then incubated for 10 min at 95 °C, and centrifuged at 18,407 g for 10 min after cooling down to room temperature. 20 μL of each sample was directly added to the middle of 180 μL 0.05 M Iodoacetamide (IAA)/100 mM Tris/HCl pH 8.0+8 M urea (UT) in a low binding Eppendorf tube and incubated for 10 min while mildly shaking at room temperature. All of the sample was transferred to a Pall 3 K omega filter (10–20 kDa cutoff, OD003C34; Pall, Washington, NY, USA) and centrifuged at 15,871 g for 30 min. Three repeated centrifugations at 15,871 g for 30 min were carried out after adding three times 100 μL UT. After that, 110 μL 0.05 M NH4HCO3 in water (ABC) was added to the filter unit and the samples were centrifuged again at 15,871 g for 30 min. Then, the filter was transferred to a new low-binding Eppendorf tube. 100 μL ABC containing 0.5 μg trypsin was added followed by overnight incubation at room temperature. Finally, the sample was centrifuged at 15,871 g for 30 min, and 3.5 μL 10% trifluoroacetic acid (TFA) was added to the filtrate to adjust the pH value of the sample to around 2. These samples were ready for dimethyl labeling.

### 2.3 Dimethyl labeling

The trypsin digested samples of pooled milk serum from each individual mother collected at several time points were labeled with light reagent (the mix of CH_2_O and cyanoborohydride), whereas trypsin digested samples of milk serum collected at each time point of each individual mother were labeled with heavy reagent (the mix of CD_2_O and cyanoborohydride). The dimethyl labeling was carried out by on-column dimethyl labeling as described previously (references). The pairs of light dimethyl label and heavy dimethyl label were then mixed up and the volume was adjusted to exactly 100 μL by adding 1 mL/L HCOOH. These samples were analyzed by LC–MS/MS [5].

The MS/MS spectra were analyzed using the Maxquant (v1.3.0.5) software package and the Andromeda search engine as previously described [6]. The function of the identified proteins was extracted from the UniprotKB database (http://www.uniprot.org/, release June 2013). The analysis resulted in the identification of 247 proteins. Of these, 21 proteins were significantly changed over lactation. The full list of quantified proteins, number of identified peptides, ratios, sequence coverage and intensities of peptides as iBAQ numbers is presented in the supplementary Excel file.

## Appendix A. Supporting information

The mass spectrometry proteomics data have been deposited to the ProteomeXchange Consortium [2] via the PRIDE partner repository with the dataset identifier PXD003465.

An Excel document containing raw as well as filtered and annotated output from MaxQuant.
